# Diagnosis of leptospira by metagenomics next-generation sequencing with extracorporeal membrane oxygenation support: a case report

**DOI:** 10.1186/s12879-023-08793-w

**Published:** 2023-11-13

**Authors:** Jianyu Ji, Wei Wang, Shulin Xiang, Xiutian Wei, Guangbao Pang, Huirong Shi, Jinda Dong, Jing Pang

**Affiliations:** 1 Research Center of Communicable and Severe Diseases, Department of Intensive Care Unit, Guangxi Academy of Medical Sciences, The People’s Hospital of Guangxi Zhuang Autonomous Region, Nanning, Guangxi China; 2 Guangxi Health Commission Key Laboratory of Diagnosis and Treatment of Acute Respiratory Distress Syndrome, Guangxi Academy of Medical Sciences, Nanning, Guangxi China; 3 Guangxi Clinical Research Center Construction Project for Critical Treatment of Major Communicable Diseases, Guangxi Academy of Medical Sciences, Nanning, Guangxi China; 4 Department of blood transfusion, The People’s Hospital of Guangxi Zhuang Autonomous Region, Nanning, Guangxi China

**Keywords:** Leptospirosis, Metagenomics next-generation sequencing, Extracorporeal membrane oxygenation, Multi-organ failure

## Abstract

**Background:**

Leptospirosis is an infectious disease caused by pathogenic *Leptospira* spp., which could result in severe illnesses. Indirect contact with these pathogens is more common. Individuals could contract this disease through contact with contaminated water or during floods. In this case, we present the details of a 40-year-old male pig farmer who suffered from severe pulmonary hemorrhagic leptospirosis and multiple organ failure. The diagnosis of leptospirosis was confirmed through metagenomics next-generation sequencing (mNGS) while the patient received extracorporeal membrane oxygenation (ECMO) support, and antibiotic treatment was adjusted accordingly. The patient underwent comprehensive treatment and rehabilitation in the intensive care unit.

**Conclusion:**

This case illustrates the importance of early diagnosis and treatment of leptospirosis. While obtaining the epidemiological history, second-generation metagenomics sequencing was utilized to confirm the etiology. The prompt initiation of ECMO therapy provided a crucial window of opportunity for addressing the underlying cause. This case report offers valuable insights for diagnosing patients with similar symptoms.

**Supplementary Information:**

The online version contains supplementary material available at 10.1186/s12879-023-08793-w.

## Introduction

Leptospirosis is a severe systemic infectious disease caused by *Leptospira *spp. Early clinical manifestations of leptospirosis lack specificity, making early clinical diagnosis challenging. Severe pulmonary leptospirosis is characterized primarily by severe pulmonary hemorrhage syndrome (SPHS) and acute respiratory distress syndrome (ARDS). This disease progresses rapidly and could lead to severe respiratory failure and life-threatening conditions before effective treatment [1]. Therefore, a prompt and accurate diagnosis and a comprehensive treatment plan based on etiology are of utmost importance. Advances in medical science have led to the development of various diagnostic methods for leptospirosis. Presently, the primary confirmatory diagnostic techniques for leptospirosis include the serological microagglutination test (MAT) and molecular-based polymerase chain reaction (PCR) [2]. In resource-limited medical centers, the availability of these tests is limited, and diagnostic accuracy is frequently inconsistent, which could be further complicated by cross-reactions with other infections [1]. In recent years, metagenomics next-generation sequencing (mNGS) has emerged as an advanced diagnostic method crucial in diagnosing challenging infections due to its unbiased and comprehensive coverage. Numerous studies have demonstrated the efficacy of mNGS in diagnosing various pathogens, including bacteria, mycoplasma, chlamydia, spirochetes, rickettsia, fungi, viruses, and parasites [3–5]. mNGS has found widespread use in clinical settings to enhance diagnostic accuracy for clinicians [6]. Extracorporeal Membrane Oxygenation (ECMO) represents an advanced life-support technology designed to replace cardiopulmonary function, making it suitable for patients with severe cardiopulmonary failure. ECMO offers a critical window of opportunity for achieving a precise diagnosis and initiating further treatment [7, 8]. In this case report, we report a case where ECMO intervention was employed early to replace and prolong the patient’s cardiopulmonary function. The diagnosis of leptospirosis was confirmed through metagenomic second-generation sequencing, leading to a comprehensive treatment plan based on the etiology to improve the patient’s prognosis.

## Case presentation

The patient is a 40-year-old male pig farm worker admitted to the hospital on October 8, 2022, with a chief complaint of “fever for 4 days.“ The fever began on October 4, 2022, with no apparent trigger, and the patient experienced a peak body temperature of 40℃. He also reported general fatigue and discomfort, unresponsive to self-administered oral ibuprofen.On October 7, 2022, the patient noticed mild jaundice and absence of petechiae. He sought medical attention at the Fever Clinic of the People’s Hospital of Guangxi Zhuang Autonomous Region, where he received ibuprofen for its antipyretic effect and ceftriaxone. On the morning of October 8, 2022, the patient’s condition worsened, marked by the onset of asthma and dyspnea, leading to his admission to the Intensive Care Unit (ICU). The patient had no significant medical history, surgical procedures, trauma, or known drug or food allergies. His vital signs upon admission were as follows: height 168 cm, weight 60 kg, body temperature 39℃, heart rate 149 bpm/min, respiratory rate 32 bpm/min, blood pressure 106/49 mmHg (with norepinephrine maintenance at 0.15 µg/kg/min). The patient appeared restless, unconscious, and uncooperative during the physical examination. Jaundice was moderately evident in the sclera of his eyes, and respiratory distress was pronounced, with thick lung sounds and prominent wet rales. Laboratory findings upon admission included a white blood cell count of 10.61 * 10^9^/L, hemoglobin 67 g/L, and platelet count of 52 * 10 ^9^/L. Coagulation function: plasma prothrombin time was measured at 15.90 s, with a PT international standardized ratio of 1.24. Plasma fibrinogen levels were 9.47 g/L. Activated partial thromboplastin time was recorded as 50.10 s, while plasma D-dimer levels were found to be 0.58 mg/L. The electrocardiogram indicated sinus tachycardia. Lung computed tomography (CT) findings included the following: (1) presence of solid and ground glass nodules in both lungs, suggestive of inflammatory nodules; (2) evidence of bilateral lung inflammation and emphysema with bullae; (3) bilateral pleural thickening .

Upon admission to the department, the patient presented with exacerbated asthma, noticeable irritability, and bloody sputum, and received treatment involving mechanical ventilation, sedation, analgesia, and anti-sympathetic therapy. Blood gas analysis results were as follows: pH 7.21, oxygen concentration 50.0%, partial pressure of carbon dioxide 41.00 mmHg, partial pressure of oxygen 92%, partial pressure of oxygen 76.00 mmHg, actual bicarbonate 16.40 mmol/L, whole blood residual base − 11.0 mmol/L, blood lactic acid 2.60 mmol/L, and oxygenation index 152 mmHg. The patient’s creatinine level was 152 µmol/L, aspartate aminotransferase was 49 U/L, total bilirubin was 368 µmol/L, direct bilirubin was 217 µmol/L, and indirect bilirubin was 151 µmol/L. Based on these test results, the patient was diagnosed with conditions such as severe pneumonia, respiratory failure, severe sepsis, liver function insufficiency, renal insufficiency, sepsis-related organ dysfunction, and severe anemia. Before initiating broad-spectrum antibiotic therapy (imipenem and cilastatin sodium), blood and bronchoalveolar lavage fluid samples were collected for bacterial culture and mNGS. Concurrently, plasmapheresis combined with continuous renal replacement therapy were administered alongside antibiotic therapy.

**Fig. 1:**
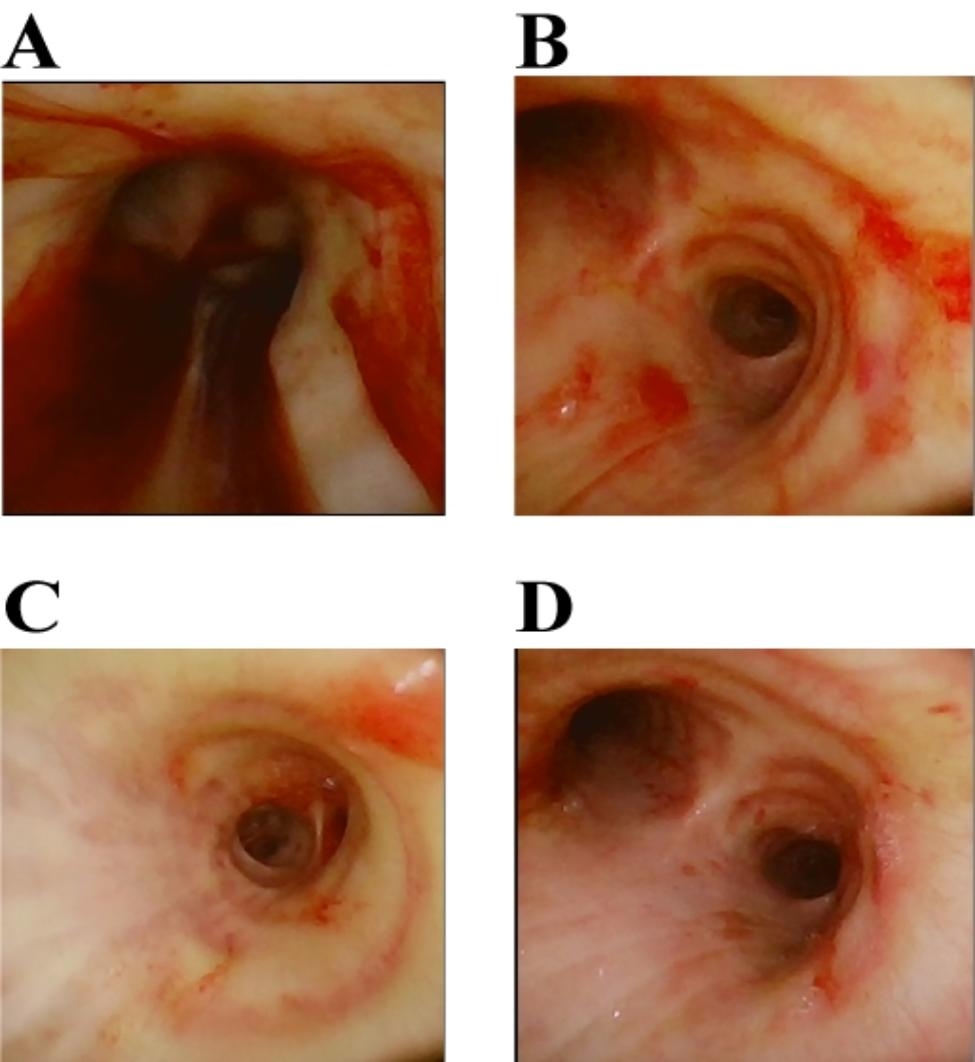
**A-B**: Bronchial images taken before treatment showing diffuse edema of the bronchial mucosa. **C-D**: Bronchial images taken after treatment showing a significant improvement in diffuse edema of the bronchial mucosa compared to before treatment

On the second day of treatment, the patient remained febrile, with a maximum temperature of 39 °C. His respiratory rate was 15 breaths/min, and blood pressure was 136/65 mmHg (with norepinephrine at 0.1 µg/kg/min). Repeat blood tests revealed a white blood cell count of 20.44 × 10^9^/L, a hemoglobin level of 63 g/L, and a platelet count of 12 × 10^9^/L. Liver function tests indicated aspartate aminotransferase at 72 U/L, total bilirubin at 328.0 µmol/L, direct bilirubin at 202.4 µmol/L, and indirect bilirubin at 125.6 µmol/L. Coagulation function results showed a plasma prothrombin time of 19.20 s, a prothrombin time (PT)-international standardized ratio of 1.65, plasma fibrinogen levels of 7.00 g/L, thrombin time of 31.50 s, and an activated partial thromboplastin time of 94.00 s. Blood gas analysis revealed a pH of 7.33, oxygen concentration of 70.0%, partial pressure of carbon dioxide of 43.0 mmHg, oxygen saturation of 95%, partial pressure of oxygen at 66.0 mmHg, and a blood lactate level of 2.40 mmol/L, with an oxygenation index of 94 mmHg. Fiberoptic bronchoscopy was conducted, yielding a substantial quantity of bloody secretions. Microscopic examination revealed diffuse congestion and edema of the airway mucosa with significant exudation (Fig. 1A-B). Intensive anti-infective therapy was initiated, combining Vancomycin with imipenem and cilastatin sodium.

**Fig. 2:**
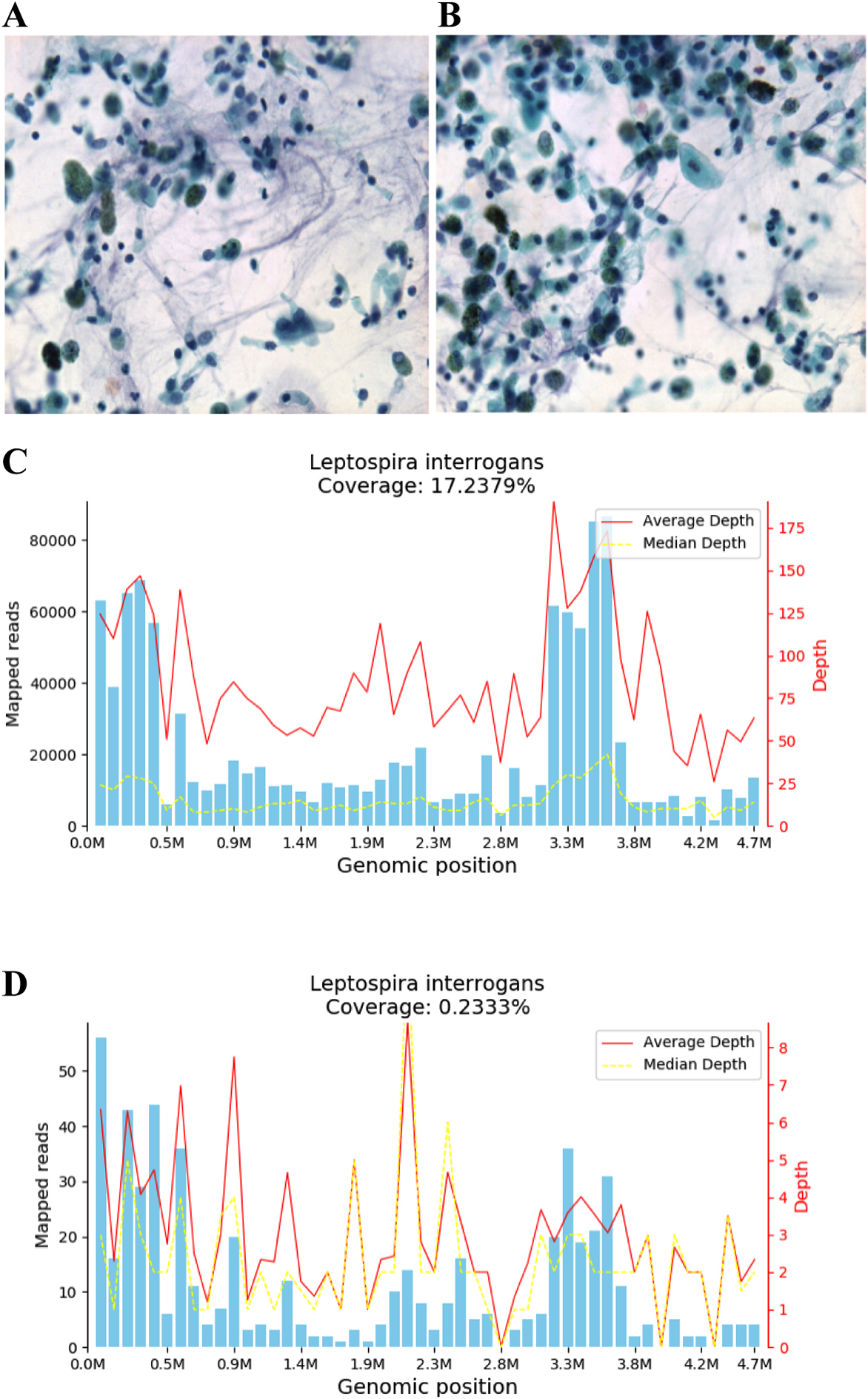
**A-B**: Cytological smear of peripheral blood; **C-D**: mNGS Results of Peripheral Blood

On the third day of treatment, peripheral blood cell morphology indicated a high white blood cell count and an elevated ratio of sorted neutrophil granulocytes, some exhibiting toxic granules and vacuolar degeneration. Various sizes of mature and heterogeneous red blood cells were observed, along with a small amount of normal platelets. No blood parasites were detected (Fig. 2A-B). Despite these findings, the patient’s respiratory failure worsened. Blood gas analysis revealed a pH of 7.38, blood oxygen concentration of 100%, carbon dioxide partial pressure of 44.00 mmHg, oxygen saturation of 86%, oxygen partial pressure of 55.00 mmHg, blood lactate level of 1.9 mmol/L, and an oxygenation index of 55 mmHg. The patient was diagnosed with severe acute respiratory distress syndrome (ARDS) and received veno-venous (V-V) ECMO support. This decision was made due to the ineffective protective and prone ventilation, consistent with the fraction of inspired oxygen (FiO2) >80% and partial pressure of arterial oxygen (PaO2)/FiO2 ratio <80 mmHg for more than 6 h [8]. At noon of the day, V-V ECMO adjuvant therapy was administered. Simultaneously, the results of metagenomics next-generation sequencing indicated a high throughput of *Leptospira *spp. sequences, with 496,403 in the blood and 1022 in the alveolar lavage fluid (Fig. 2C-D; Tables 1 and 2). Bronchoalveolar lavage fluid also revealed the presence of genera such as *Streptococcus*, *Neisseria, Corynebacterium, Dolosigranulum*, and *Olsenella*, considered bacterial contaminants as part of the saprophytic flora. Based on these findings, high-dose penicillin therapy at 3.2 million units IV every 4 h and daily dexamethasone at 5 mg were administered to manage Jarisch–Herxheimer reaction.

**Table 1:** Results of second-generation gene sequencing of the patient’s alveolar lavage fluid

Type	Genus name	Relative abundance	Number of sequences	Species Name	Identification confidence level	Number of sequences
G^−^	Leptospira interrogans	3.90%	1022	Leptospira interrogans	99%	704
G^+^	Streptococcus	66.60%	3,427	Streptococcus mitis	99%	383
				Streptococcus oralis	99%	82
G^−^	Neisseria	5.70%	238	Neisseria lactamica	99%	20
G^+^	Corynebacterium	0.50%	30	Corynebacterium propinquum	99%	7
G^+^	Dolosigranulum	0.20%	28	Dolosigranulumpigrum	99%	28
G^+^	Olsenella	0.20%	38	Olsenella uli	99%	38

**Table 2:** Patient’s blood second generation gene sequencing results

Type	Genus name	Relative abundance	Number of sequences	Species Name	Identification confidence level	Number of sequences
G^−^	Leptospira interrogans	99.40%	2,094,354	Leptospira interrogans	99%	490,430
G^+^	Micrococcus	0.1%	24	Micrococcus luteus	99%	24
virus	Polyomavirus	0.5%	385	JC polyomavirus	99%	385

**Fig. 3:**
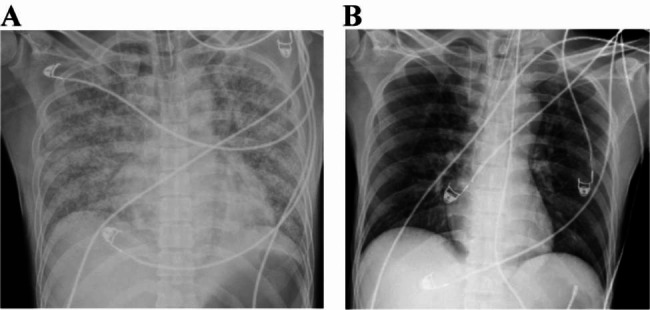
The changes in pulmonary X-ray before and after ECMO support

The patient’s condition improved significantly following comprehensive treatment in the intensive care unit (Table 3). On day 6, corresponding to the fourth day of the ECMO operation, a marked reduction in pulmonary hemorrhage and mucosal exudation was observed during fiber-optic bronchoscopy (Fig. 1C-D). On day 7, corresponding to the fifth day of the ECMO operation, the patient successfully passed the ECMO pinch withdrawal test and was transitioned from ECMO support. By day 11, the patient’s respiratory function and oxygenation had improved to the extent that they passed the spontaneous breathing trial (SBT) and were successfully weaned from ventilator support. A follow-up chest radiograph showed significant resorption of bilateral lung lesions (Fig. 3). The patient was discharged on day 21 of the disease course, and 3 months later, follow-up examinations revealed no significant abnormalities in physical parameters, with the patient resuming a normal lifestyle.

**Table 3:** Changes in blood gases, ventilator conditions and infection indicators before and after ECMO on board

Time	Emergency admissions	Before ECMO	In ECMO	At withdrawal	After withdrawal
PH	7.21	7.38	7.39	7.50	7.48
PaO_2_ (mmHg)	76	55	167	143	158
paCO_2_ (mmHg)	35	44	35	43	41
Oxygenation Index	152	55	417	318	316
FiO2 (%)	50	100	40	45	50
PEEP (cm H_2_ O)	-	12	12	10	10
VT (ml/kg)	-	6	5	8	8
PCT (ng/ml)	> 50	> 50	44.05	27.63	1.14

## Discussion

Leptospirosis is an acute systemic infectious disease primarily caused by *Leptospira* spp. It is a naturally occurring epidemic disease, mainly prevalent in tropical and subtropical regions, with a concentration in agricultural development areas [9]. Pathogenic *Leptospira* spp. could infect most mammals, although it has become relatively rare due to improved living standards and heightened health awareness among residents [10]. Following the contraction of leptospirosis, there may be a variable incubation period (typically 7–14 days). The disease presents various clinical forms, including early, intermediate, and late stages [11]. The early phase (leptospirosis septicemia, 1–3 days post-onset) is frequently described as “fever, aches and pains, red eyes, sore legs, and enlarged lymph nodes.“ In contrast, the intermediate stage (organ damage, 3–10 days post-onset) encompasses multiple clinical patterns, including influenza-like, typhoid-like, pulmonary hemorrhage, diffuse pulmonary hemorrhage, jaundice hemorrhage, renal failure, and meningoencephalitis. The late stage (post-lesion phase) encompasses post-lesion fever, ocular post-lesion, reactive meningitis, and occlusive cerebral arteritis. The clinical symptoms of leptospirosis are variable and lack typical specificity, ranging from mild cold-like symptoms to severe multi-organ failure, which makes clinical diagnosis challenging.

Upon initial admission, PCR, MAT, and smears of alveolar lavage fluid and blood samples yielded negative results. Subsequently, we consulted with the professor at the microbiology lab and conducted a second dark-field electron microscopy examination. However, no positive evidence of the etiology was found. The potential correlation might arise from the patient’s previous use of antibiotics (ceftriaxone sodium) before specimen collection and the limited capacity of the laboratory equipment. For MAT and pathogen isolation culture and PCR in the laboratory, the sample collection requirements are stringent, the process is time-consuming, and the positive rate is low. Furthermore, the laboratory departments of medical institutions have high standards, which do not facilitate the rapid diagnosis and treatment of severe leptospirosis. The illness comprises two distinct clinical phases: septicemia due to *Leptospira* spp. or the acute and immune phases [12]. Given the onset of the disease in many patients, relying solely on one method, either MAT or PCR, might result in missed cases or misdiagnosis. These factors contribute to a high mortality rate among patients with severe leptospirosis [13]. Since 2014, high-throughput metagenomic sequencing has gradually been employed to detect leptospirosis infections, offering rapid and highly accurate results [14]. In this case report, the patient underwent a conventional pathogenic examination upon admission, with alveolar lavage fluid and blood specimens retained for metagenomics next-generation sequencing. In the absence of positive results from real-time PCR, MAT, and smear tests, the metagenomics next-generation sequencing results indicated the presence of leptospira interrogans in both alveolar lavage and blood samples. The combination of conventional screening and metagenomics next-generation sequencing could significantly improve the sensitivity and serve as a valuable complement to clinical pathogenesis [15]. While mNGS has played a significant and beneficial role in etiological diagnosis, it still encounters challenges in clinical application, including the lack of standardized quality control standards and result interpretation. As mNGS technology advances, the operation will become more automated and standardized, becoming a routine diagnostic technology for identifying infectious disease etiology.

ECMO, also known as Extracorporeal Life Support (ECLS), is an extracorporeal life support technology proven highly significant in treating patients with severe respiratory and circulatory failure. This technology primarily operates through various modes of extracorporeal air-blood exchange, supporting both cardiac and pulmonary functions. Simultaneously, it facilitates the removal of carbon dioxide and enhances oxygen delivery, thereby improving tissue perfusion. ECMO offers a critical window of opportunity to stabilize vital signs and effectively treat individuals with severe cardiac and pulmonary failure by offering prolonged cardiopulmonary support [16]. In 2017, a case of leptospirosis diagnosed through serological testing and treated with a combination of ECMO-supported therapy and antibiotics. The patient recovered completely without any complications [17]. Similarly, another case of leptospirosis accompanied by acute respiratory failure and acute kidney injury. The diagnosis was confirmed through PCR DNA testing. The patient underwent treatment involving extracorporeal membrane lung support with blood perfusion and spent 19 days in the ICU (6 days on ECMO and 12 days on mechanical ventilation). Post-extubation, the patient achieved oxygen saturation of 98% while breathing in room air. Spirometry performed 2 weeks later revealed a mild restrictive breathing pattern [18]. Within China, one expert reported the diagnosis of leptospirosis confirmed by second-generation sequencing of alveolar lavage fluid. Successful treatment involved the administration of penicillin as an anti-infective agent and V-V ECMO [19].

The case initially presented with fever, jaundice, and dyspnea, although these symptoms lacked specificity. Nevertheless, they continued to deteriorate, and imaging showed progressive changes. Laboratory investigations indicated multi-organ dysfunction; fiberoptic bronchoscopy revealed the presence of large quantities of bloody secretions, diffuse congestion, and edema and exudation of the airway mucosa. Despite treatment involving broad-spectrum anti-infection measures, organ function preservation, protective mechanical ventilation, and prone positioning, the patient’s condition necessitated VV-ECMO to address the life-threatening exacerbation of respiratory failure. The timely implementation of ECMO technology facilitated adequate oxygenation during the patient’s treatment. Early identification and treatment of the underlying cause of the disease are crucial in slowing its progression, promoting recovery, and reducing the risk of complications. Initial in-hospital tests, such as real-time PCR, MAT, and smear examinations of alveolar lavage and blood samples, yielded negative results upon admission. However, high-throughput sequencing revealed leptospirosis, leading to the diagnosis of pulmonary hemorrhagic leptospirosis and severe respiratory distress syndrome. The disease progressed rapidly in its early stages, involving multiple organ insufficiencies affecting the respiratory, circulatory, hepatic, renal, and coagulation systems [20]. Following adjustments to the anti-infection regimen, intensive rehabilitation, and nutritional support, the patient’s condition gradually improved, eventually resulting in discharge from the hospital.

In summary, early identification of leptospirosis can be challenging, and patients are susceptible to progressing to a severe, life-threatening form of the disease. A combination of routine clinical screening and metagenomics next-generation sequencing allows for rapid identification and diagnosis. Early identification, diagnosis, etiological treatment, dynamic assessment, and standardized comprehensive treatment might reverse the disease’s progression. If conventional therapy fails to maintain adequate respiration and circulation, ECMO could be considered after a thorough assessment, providing a valuable window before effective treatment occurs.

### Electronic supplementary material

Below is the link to the electronic supplementary material.

Supplementary Material 1

Supplementary Material 2

Supplementary Material 3

## Data Availability

All data have been presented within the manuscript and in the form of images.
