# Supplementary figures and images for: Diagnosis of leptospira by metagenomics next-generation sequencing with extracorporeal membrane oxygenation support: a case report
Source: BMC Infect Dis. 2023 Nov 13;23:788. doi: 10.1186/s12879-023-08793-w (PMC10644436; doi:10.1186/s12879-023-08793-w)

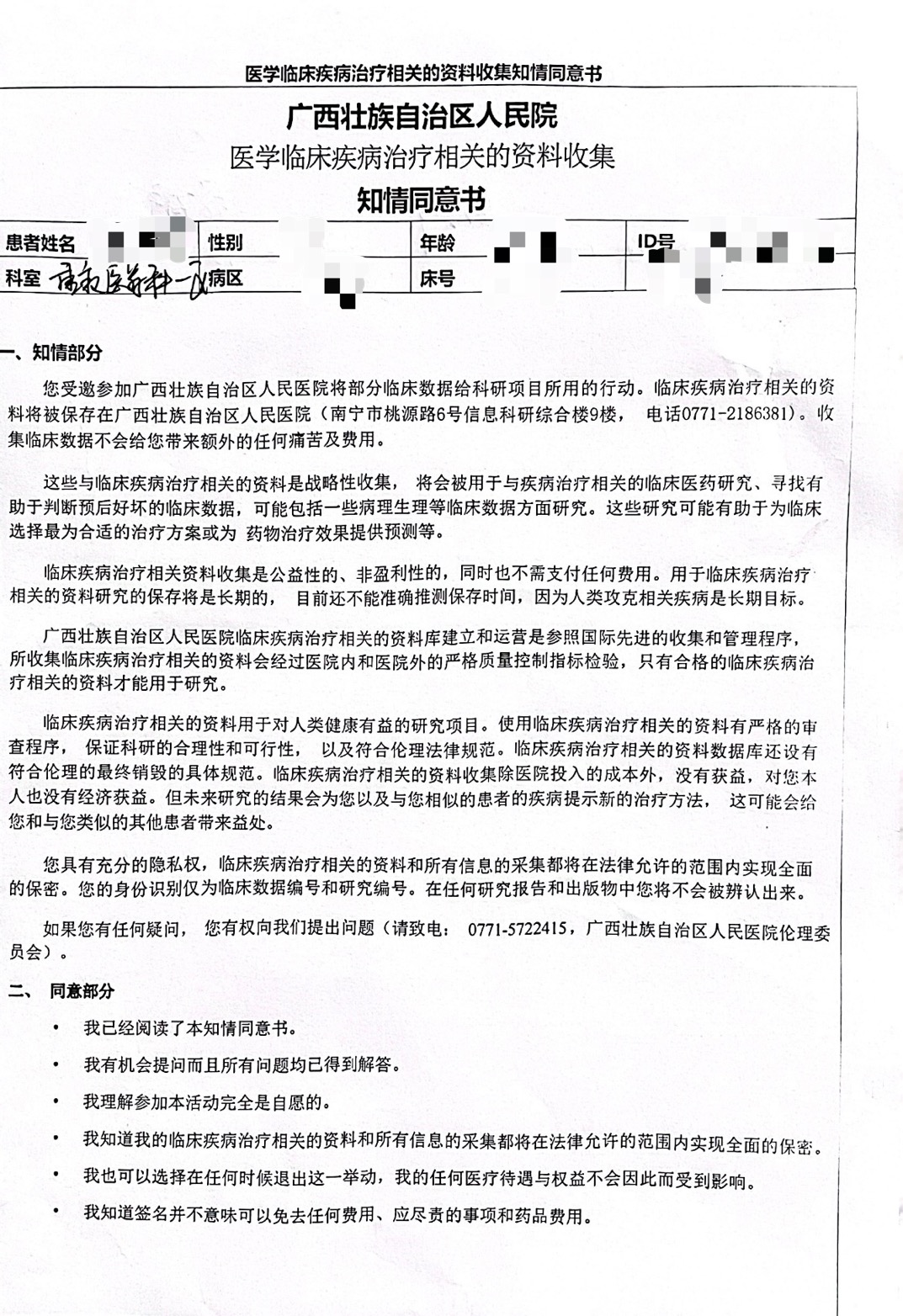

Supplement: Supplementary file 1 — Supplementary Material 1 [file 12879_2023_8793_MOESM1_ESM.jpg]

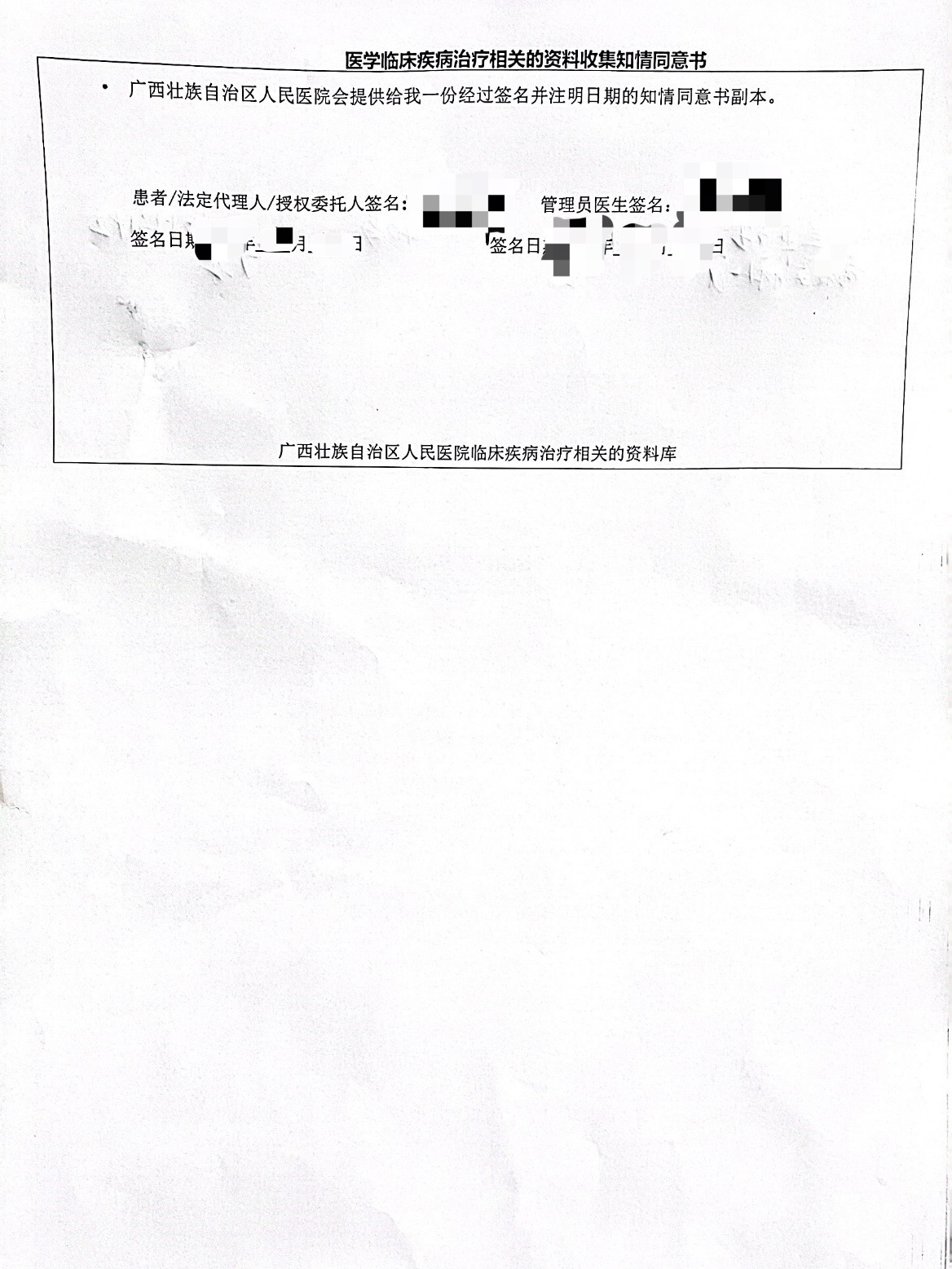

Supplement: Supplementary file 2 — Supplementary Material 2 [file 12879_2023_8793_MOESM2_ESM.jpg]
